# Environmental Changes During the Late Glacial and Early Holocene Transition Revealed by Palaeolimnological Record from Southern Lithuania

**DOI:** 10.3390/biology15060499

**Published:** 2026-03-20

**Authors:** Gražyna Kluczynska, Neringa Gastevičienė, Vaida Šeirienė

**Affiliations:** State Scientific Research Institute Nature Research Centre, Akademijos 2, LT-08412 Vilnius, Lithuania; grazyna.kluczynska@gamtc.lt (G.K.); neringa.gasteviciene@gamtc.lt (N.G.)

**Keywords:** Cladocera, diatoms, plant macrofossils, Paleoecology, lake sediments

## Abstract

This study presents integrated Cladoceran, plant macrofossil and diatom-based environmental reconstruction from the Čepkeliai Bog (Southern Lithuania), covering the Late Glacial–Early Holocene transition. The objective was to assess palaeoenvironmental changes with a focus on trophic state, acidification, and water-level fluctuations and to explore the applicability of these assemblages as temperature-sensitive indicators. The findings of our study revealed that sedimentation started at about 13,200–13,000 cal yr BP in a deep, oligotrophic and cold-water palaeobasin. Inferred palaeoenvironment changes correlate with the GI-1b event (Gertsenzee oscillation). A significant ecological shift to a shallow, warm, ecologically diverse environment occurredat about 13,000 cal yr BP and is consistent with the GI-1a (Allerød) period. The Younger Dryas (12,850–11,650 cal yr BP) is characterised by a rise in lake level and oligo-mesotrophic and high-water transparency conditions. At the end of the Younger Dryas (around 12,000 cal yr BP), climate warming and a drop in water levels were recorded. Intensive palaeobasin swamping processes began around 9700 years cal yr BP, during the Boreal period. A short-lived “9.2” cooling event was fixed at about 9200–9000 cal yr BP. The results obtained provide new insights into postglacial palaeoenvironmental dynamics in the southeastern Baltic region.

## Simple Summary

Currently, climate change has a significant impact on biodiversity. In order to accurately assess and predict the extent of these changes and the factors that caused them, as well as future trends, it is necessary to understand how these processes have unfolded over a longer period of time. This research focuses on the transition from the Late Glacial period to the Early Holocene, a period characterised by short-term and rapid climate changes similar to those occurring today. Three biotic proxies—Cladoceran, plant macrofossil and diatom analysis—are used in this study, which increases the reliability of the environmental reconstructions. Our results show that sedimentation started around 13,200–13,000 cal yr BP in a deep, oligotrophic palaeobasin. At about 13,000 cal yr BP, Allerød climatic warming influenced the shift to a shallow, ecologically diverse environment. During the Younger Dryas (12,850–11,650 cal yr BP), a rise in lake level and oligo-mesotrophic high water transparency conditions were fixed. At around 12,000 cal yr BP, climate warming and a drop in water levels were recorded. Intensive palaeobasin swamping processes began around 9700 cal yrs BP (Boreal).

## 1. Introduction

Short-term ecological studies are insufficient for making reliable predictions about the potential consequences of future environmental changes. To predict the nature and extent of future biotic responses to environmental change, it is necessary to understand the complexity of the system’s response to these changes at different time scales [1]. The transitional period from the Late Glacial to the Early Holocene experienced rapid and short-lived climate changes which could be considered analogous to those predicted for the present century. Particularly, the rapid warming trend at the end of the Younger Dryas and the beginning of the Holocene [2,3] has been proposed as a possible past climate analogue because both the magnitude and rates of change parallel those predicted for the present century [4]. To better understand the complex natural processes of this time period, we need the most accurate regional data available. Although more detailed studies of lake sediment records from this period have recently been conducted in the northeastern Baltic region [5,6,7,8,9,10,11], further research is necessary to improve regional palaeoenvironmental reconstructions. Since the vast majority of studies are based on pollen analysis, biological proxies such as diatoms and cladocerans can provide valuable information on past changes in lake levels, trophic status, and the structure of the aquatic ecosystem. A large variety of biotic and abiotic approaches have been exploited from sedimentary archives and used to reconstruct past environments. Cladoceran and diatom assemblages are among the most frequently applied biological proxies in palaeolimnological reconstructions, owing to their high sensitivity to environmental changes and excellent preservation in lake sediments [11,12,13,14,15,16,17]. Cladocerans are small planktonic and benthic freshwater crustaceans whose chitinous remains, particularly head shields, postabdomens, and claws, are often well preserved in sedimentary archives, allowing high-resolution taxonomic identification [18,19,20]. The ecological preferences of cladoceran assemblages span key limnological parameters such as temperature, trophic status, pH, macrophyte abundance and hydrological regime. This makes them a well-established proxy for past environmental changes in lakes [21,22,23,24,25,26,27].

Diatoms—siliceous unicellular algae—are likewise highly sensitive indicators of past aquatic environments. Their short life cycles, high reproductive rates, and narrow ecological tolerances make them ideal for detecting changes in pH, salinity, nutrient concentrations, water depth, and temperature-related hydrological variability [28,29,30]. Fossil diatom assemblages have been widely used in lake and peatland records to infer acidification, eutrophication, and hydrological shifts [12,13,14,31,32,33]. The complementary use of these two microfossil groups enhances the reliability of palaeoenvironmental interpretations because of their differing ecological niches and taxonomic resolution.

Here, we present environmental reconstructions based on cladoceran, diatom and plant macrofossil studies of the Čepkeliai Bog (southern Lithuania) sediment section deposited during the Late Glacial–Early Holocene period. The goal of this study is to evaluate the palaeoenvironmental changes, with a primary focus on the trophic state, dissolved mineral salts, and water-level fluctuations, as well as to explore how these communities respond to climate change. To date, no palaeoecological studies based on Cladocera have been conducted in Lithuania. This investigation addresses that gap, offering a novel contribution to regional palaeolimnology. Diatom assemblages are presented alongside Cladocera remains, providing an additional line of palaeolimnological evidence that enhances the interpretation of environmental dynamics recorded in the Čepkeliai Bog sediments. Supporting multi-proxy datasets, comprising pollen, plant macrofossils, loss-on-ignition, magnetic susceptibility, and geochemical data spanning the Holocene, have been previously reported by Stančikaitė [34] and serve here as a contextual framework for interpreting our biological proxy data. We hope that our studies will expand our understanding on postglacial palaeoenvironment dynamics in the southeastern Baltic region.

## 2. Materials and Methods

### 2.1. Study Site

**Figure 1:**
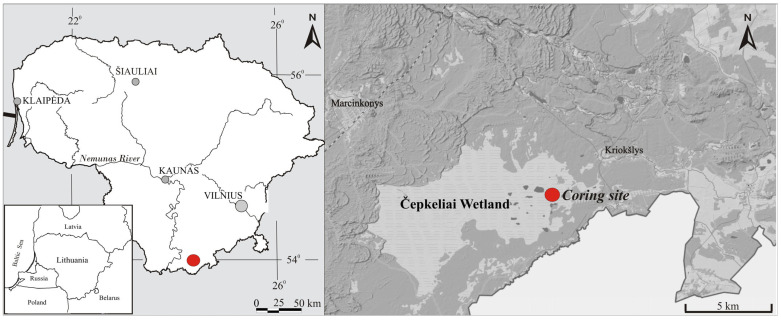
Location of the study site. The coring site is shown by a red circle.

This study was conducted within the Čepkeliai wetland complex, located in southeastern Lithuania near the Belarusian border (54°00′48.54″ N; 24°37′01.02″ E). Covering more than 5858 ha, Čepkeliai is one of the largest and best-preserved peatland systems in both Lithuania and Northern Europe. The sampling site lies in the southeastern sector of the bog (54°00′ N; 24°30′ E), approximately 2.5 km from the village of Kriokšlys, at an elevation of ~131 m above sea level, within gently undulating terrain ranging from 128.5 to 134.4 m a.s.l. (Figure 1). The region is situated in the boreo-nemoral biogeographical zone. The surrounding forest is predominantly composed of *Pinus sylvestris* L., *Picea abies* (L.) Karst., *Betula pubescens* Ehrh., and *Betula pendula* Roth [35]. The wetland landscape forms a diverse habitat mosaic, including raised bogs, sedge communities, alder swamps, dry pine heaths, and shallow lakes. Due to minimal anthropogenic disturbance, the Čepkeliai bog represents a near pristine peatland ecosystem. The regional climate is moderately continental. The mean annual temperature is 6.2 °C, with average annual precipitation of 673 mm. July is typically the warmest month (17.7 °C) and January the coldest (−5.4 °C). Snow cover generally persists for 75–80 days per year, and the area experiences some of the highest diurnal temperature amplitudes in Lithuania. These climatic parameters are based on long-term meteorological records from the Varėna weather station, operated by the Lithuanian Hydrometeorological Survey.

### 2.2. Coring and Sampling

Sediment cores were obtained from the southeastern sector of the Čepkeliai Bog using a Russian-type peat corer equipped with a 5 cm diameter inner chamber. The lithology of sediment sequences was described visually in the field, wrapped into plastic tubes and transported to the laboratory for subsampling. The sampling encompassed a depth interval of 700–1500 cm.

### 2.3. Loss-on-Ignition

To assess organic matter content, loss-on-ignition (LOI) analysis was conducted following established methodologies [36]. Sediment samples, each approximately 2 cm thick, were taken at an average interval of 6 cm. Samples were combusted at 550 °C, and the results are presented as percentages of organic matter by dry weight.

### 2.4. Chronostratigraphy

Twelve radiocarbon dates from the Holocene section of the Čepkeliai sediment sequence have been published previously [34]. To constrain the chronology of the deeper Late Glacial sediments analysed in this study, an additional four bulk samples were dated using radiocarbon (^14^C) analysis. Measurements were carried out at the Laboratory of Nuclear Geophysics and Radioecology, Nature Research Centre, Vilnius. Calibration was performed using the IntCal20 calibration curve [37] in OxCal v4.4 [38].

### 2.5. Cladocera Analysis

Cladocera analysis was performed on 112 sediment samples, each with a volume of 1 cm^3^. Sample preparation followed the standard method of Frey [18]. Carbonate material was removed by treatment with 10% HCl, and organic matter was digested by heating the samples in 10% KOH. The residue was sieved through a 33 µm mesh and rinsed with distilled water. A 0.1 mL aliquot of each suspension was mounted on a microscope slide for analysis. A minimum of 200 Cladocera remains per sample, including head shields, valves, postabdomens, claws, and ephippia, were identified using a light microscope at magnifications of ×10, ×20, and ×40. Taxonomic identification followed Szeroczyńska and Sarmaja-Korjonen [20]. The results are presented as a percentage diagram created using TILIA (version 1.0.1) [39], with final editing in CorelDRAW X5. Cluster analysis was performed using CONISS (stratigraphically constrained incremental sum of squares) [40]. The analysis was based on percentage data of all identified Cladocera taxa, without transformation. Zonal boundaries were defined based on major changes in the stratigraphically constrained dendrogram, and eight local Cladocera assemblage zones (LCAZ) were distinguished. Species were categorised into planktonic and littoral functional groups [20].

### 2.6. Diatom Analysis

Diatoms were extracted following standard procedures outlined by Battarbee [41] and Miller and Florin [42]. Carbonate material was removed using hydrochloric acid (HCl), and organic matter was oxidised with 30% hydrogen peroxide (H_2_O_2_). Clay and mineral particles were separated by decantation and heavy liquid flotation using a mixture of cadmium iodide (CdI_2_) and potassium iodide (KI). Cleaned diatom material was mounted in NBS Naphrax (refractive index = 1.74) and analysed under a light microscope at 1000× magnification with oil immersion. Species identification was based primarily on Krammer and Lange-Bertalot [43,44,45,46]. A minimum of 500 diatom valves were counted per slide. Taxa were classified according to ecological preferences following Hustedt [47] and Van Dam [48]. The most abundant and ecologically significant taxa are presented as percentages of the total valve count. Diatom data were visualised using TILIA software (version 1.2) [49].

### 2.7. Plant Macrofossil Analysis

Plant macrofossil analysis was carried out on the deeper part of the sediment sequence, corresponding to the Late Glacial period. The analysed section represents a stratigraphic continuation of the overlying Holocene deposits previously studied and published by Stančikaitė [34], allowing for an extended palaeoenvironmental reconstruction. Sediment samples of 39 cm^3^ were collected at 4 cm intervals. Each sample was wet-sieved using a 0.2 mm mesh to extract plant remains. Macrofossils were examined under a NIKON SMZ 1500 stereomicroscope (Nikon Europe B.V., Amstelveen, The Netherlands), and taxonomic identification followed the procedures described by Grigas [50], Berggren [51,52], and Cappers [53]. Plant nomenclature was based on the classification system proposed by Gudžinskas [54]. Macrofossil data were visualised using TILIA [39], and final editing of the diagram was performed in CorelDRAW X5. The results are presented as absolute counts. Local macrofossil assemblage zones (LMAZ) were defined using CONISS cluster analysis (stratigraphically constrained incremental sum of squares) [40]. The analysis was performed on macrofossil count data, and zonal boundaries were defined based on major changes in the stratigraphically constrained dendrogram.

## 3. Results

### 3.1. Lithology and Loss-on-Ignition (LOI)

**Figure 2:**
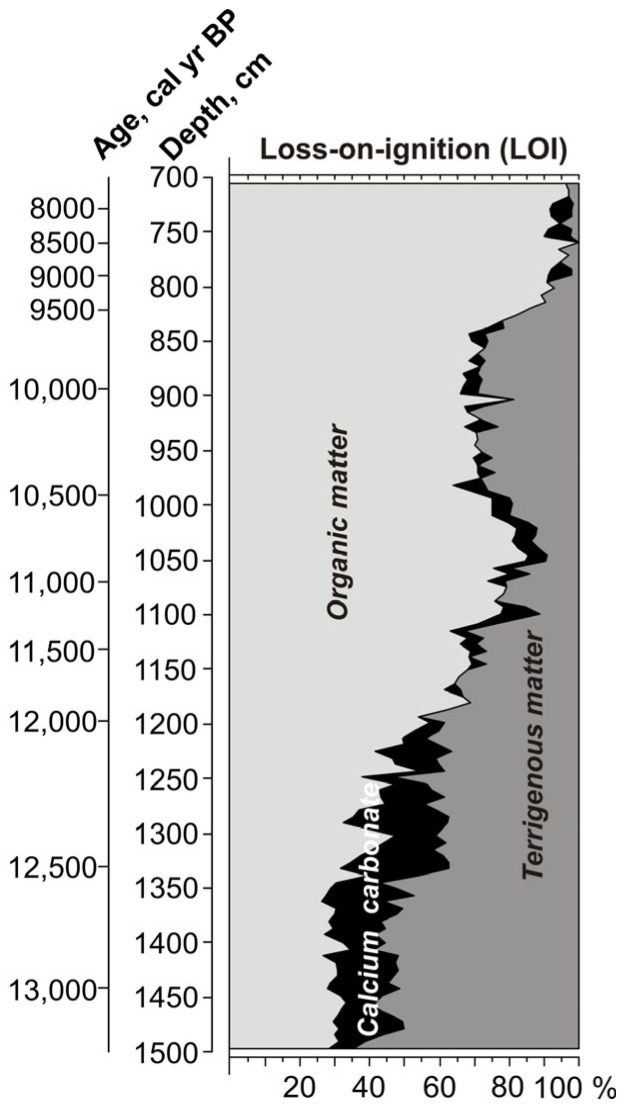
Loss-on-ignition (LOI) diagram.

Lithological description of the section was based on visual inspection of the core sediments (Table 1). The relative amounts of organic matter, calcium carbonate, and terrigenous material were estimated using loss-on-ignition (LOI). The proportions of organic matter, calcium carbonate, and terrigenous material obtained from LOI analysis are shown in Figure 2.

The analysed sediment sequence is composed predominantly of different types of gyttja (Table 1; Figure 2). The lowermost part of the profile (1500–1410 cm) consists of sandy black gyttja and is characterised by a high proportion of terrigenous matter (ca. 50–65%), relatively low organic matter content (ca. 27–33%), and CaCO_3_ values ranging between 7% and 21%. This unit is overlain by black gyttja (1410–1370 cm), where terrigenous matter remains relatively abundant (ca. 52–59%) while organic matter slightly increases (ca. 27–35%).

Above this interval, dark greenish-grey silty gyttja with black layers occurs between 1370 and 1300 cm. In this interval, organic matter ranges between 26% and 40%, CaCO_3_ varies between 12% and 31%, and terrigenous matter between 37% and 57%. Between 1300 and 1200 cm the sediments are represented by dark greenish-grey silty gyttja, where organic matter reaches up to 55% and terrigenous matter ranges between 36% and 53%, indicating more stable sedimentation conditions within the basin.

The overlying part of the sequence corresponds to the interval previously described by Stančikaitė [34]. In this interval, silty gyttja between 1100 and 1200 cm is characterised by relatively high terrigenous matter content exceeding 47% and organic matter values ranging between 54% and 71%, indicating unstable sedimentation conditions. The overlying gyttja (1050–1100 cm) shows a marked increase in organic matter content (up to 85%) accompanied by a decrease in terrigenous matter. Similarly high organic matter values (85%) were recorded in the interval 1034–1050 cm, reflecting increasing biogenic productivity and stabilisation of the sedimentation regime. Above 1034 cm, terrigenous matter becomes more abundant, indicating the onset of a new stage in the sedimentation history. This interval is followed by the accumulation of organic-rich gyttja in the upper part of the sequence [34].

### 3.2. Chronology

The sediment sequence between 700 and 1500 cm corresponds to a calibrated radiocarbon age range of approximately 13,200–7650 cal yr BP. All radiocarbon dates used in this study are presented in Table 2. The age–depth model was constructed using the radiocarbon dates reported by Stančikaitė [34] together with four additional dates obtained from deeper intervals of the sequence.

**Figure 3:**
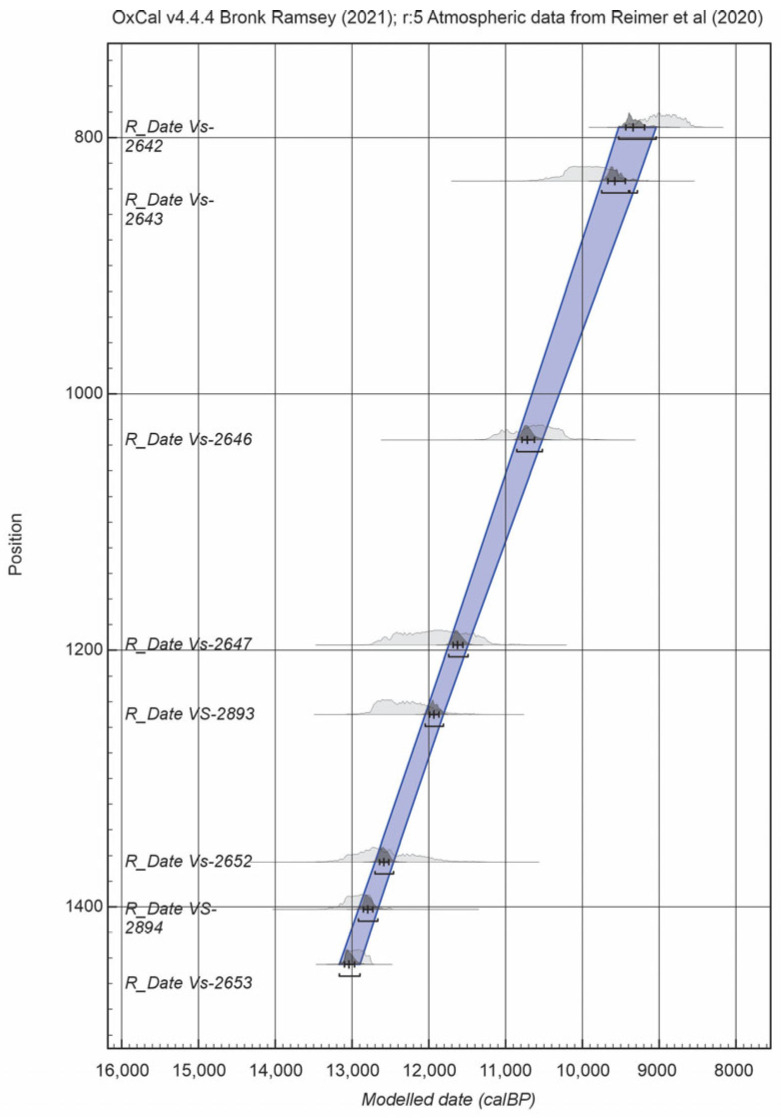
Age–depth model for the Čepkeliai section [37,38].

The age–depth model (Figure 3) indicates that sedimentation in the basin started at approximately 13,200–13,000 cal yr BP. The earliest phase of sedimentation is characterised by minerogenic-rich deposits, suggesting unstable sedimentary conditions. A gradual increase in organic matter indicates stabilisation of the sedimentary environment until approximately 10,900 cal yr BP [34]. Subsequently, increased terrigenous input reflects renewed instability in the sedimentation regime until about 9200 cal yr BP, followed by the accumulation of predominantly organic-rich sediments in the upper part of the sequence [34].

### 3.3. Cladocera Assemblages

**Figure 4:**
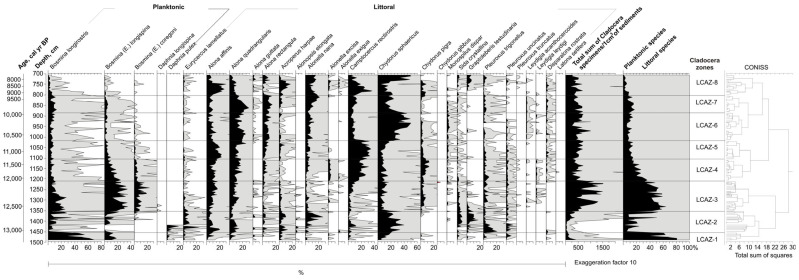
Relative abundance (%) diagram of subfossil cladoceran taxa in the Čepkeliai section. Black areas represent the original percentage values, while grey shading indicates exaggeration curves used to highlight low-percentage taxa.

The subfossil Cladocera fauna is represented by 29 taxa assigned to four families: Chydoridae (22 taxa), Bosminidae (3 taxa), Daphniidae (2 taxa), and Sididae (2 taxa). Planktonic species are represented primarily by Bosminidae, while littoral taxa are dominated by members of Chydoridae and Sididae. Based on changes in species composition and abundance, eight local Cladocera assemblage zones (LCAZ) were distinguished (Figure 4).

LCAZ-1 (1489–1457 cm): Among planktonic taxa, *Bosmina longirostris* dominates (up to 70.1%), followed by *Bosmina longispina* (18.5%) and *Daphnia pulex* (3.8%). The most abundant littoral species is *Chydorus sphaericus* (18.6%).

LCAZ-2 (1457–1355 cm): Littoral taxa dominant (up to 95.5%), including *Acroperus harpae*, *Alonella nana*, *Chydorus sphaericus*, *Eurycercus lamellatus*, *Graptoleberis testudinaria*, and *Sida crystallina*. Planktonic taxa decline markedly; *Bosmina longispina* disappears at the base of the zone. *Daphnia pulex* is initially frequent but disappears toward the top, where *Bosmina longispina* and *Bosmina* (*E.*) *coregoni* reappear. *Pleuroxus trigonellus* and *Eurycercus lamellatus* decrease in the upper part of the zone, whereas *Graptoleberis testudinaria*, *Sida crystallina*, and *Alonella nana* increase.

LCAZ-3 (1355–1205 cm): Planktonic species become dominant (up to 65%), including *Bosmina* (*E.*) *longispina*, *Bosmina* (*E.*) *coregoni*, and *Bosmina longirostris*. The abundance of littoral taxa such as *Alona rectangula*, *Acroperus harpae*, *Alonella nana*, *Chydorus sphaericus*, and *Graptoleberis testudinaria* decreases. *Chydorus piger* appears for the first time.

LCAZ-4 (1205–1111 cm): The zone is marked by an increase in *Camptocercus rectirostris*, *Chydorus piger*, *Alona excisa*, and *Alona rectangula*. *Disparalona rostrata* appears, whereas *Alonopsis elongata* disappears. The abundance of *Bosmina* (*E.*) *longispina*, *Bosmina* (*E.*) *coregoni*, and *Bosmina longirostris* declines.

LCAZ-5 (1111–1011 cm): *Camptocercus rectirostris* dominates (up to 34.6%), followed by *Alona quadrangularis* (24.8%), *Alona affinis* (23.1%), *Chydorus sphaericus* (17.5%), *Alonella nana* (7.2%), *Alona rectangula* (8.5%), *Acroperus harpae* (9.0%), and *Pleuroxus trigonellus* (8.5%). *Chydorus piger* declines in abundance. Planktonic taxa are scarce: *Bosmina* (*E.*) *longispina* (2.5%), *Bosmina longirostris* (1.9%), and *Bosmina* (*E.*) *coregoni* (0.5%). *Alona excisa* disappears, while *Monospilus dispar* becomes more frequent.

LCAZ-6 (1011–887 cm): *Chydorus sphaericus* becomes dominant (up to 50.5%). *Camptocercus rectirostris* declines significantly to 3%), whereas *Alona rectangula* (13.4%), *Alonella nana* (12.9%), and *Monospilus dispar* (4%) increase. *Acroperus harpae* shows a decreasing trend.

LCAZ-7 (887–805 cm): *Chydorus sphaericus* decreases to 10.4%, while *Alona quadrangularis* (29.4%), *Alona rectangula* (15%), and *Alonella nana* (21.2%) increase. *Acroperus harpae*, *Pleuroxus trigonellus*, and *Sida crystallina* become more abundant. Planktonic taxa show a slight increase.

LCAZ-8 (805–707 cm): This zone is characterised by peaks in *Camptocercus rectirostris* (36.4%), *Alona affinis* (30%), and *Acroperus harpae* (17.5%). *Alona quadrangularis* (10.5%), *Alona rectangula* (0.5%), and *Alonella nana* (5%) show lower abundances. *Alonella exigua*, *Alona excisa*, *Graptoleberis testudinaria*, and *Chydorus piger* increase. Planktonic taxa decline sharply. At approximately 711 cm, *Chydorus sphaericus* shows a sharp peak (50.2%), whereas *Camptocercus rectirostris*, *Alona affinis*, *Acroperus harpae*, *Alonella exigua*, and *Alona excisa* decrease significantly. *Alona rectangula*, *Pleuroxus trigonellus*, and *Sida crystallina* increase in abundance.

### 3.4. Diatom Assemblages

**Figure 5:**
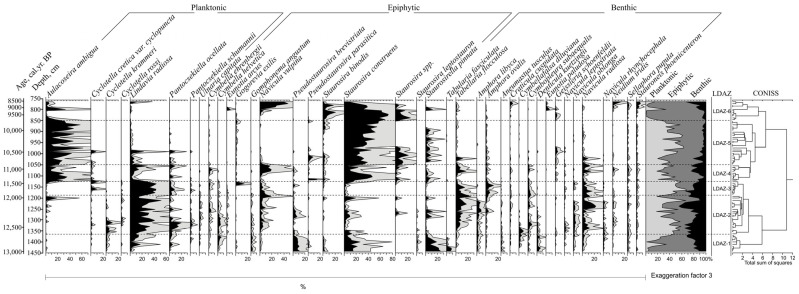
Percentage diatom diagram of the most abundant diatom taxa in the Čepkeliai section. Black areas represent the original percentage values, while grey shading indicates exaggeration curves used to highlight low-percentage taxa.

Rich diatom flora consisting of 92 species, belonging to 27 genera, was found in the studied sediments. According to compositional changes in identified species throughout the section, six local diatom zones (LDAZ) were distinguished (Figure 5).

LDAZ-1 (1450–1352 cm): The zone is dominated by epiphytic taxa comprising about 85%, while benthic taxa vary from about 10 to 20% and planktonic from about 5 to 25%. Small *Staurosira* and *Pseudostaurosira* species prevail among the epiphytic taxa. Plankton is dominated by oligotrophic *Lindavia radiosa* and *Pantoseckiella ocellata*.

LDAZ- 2 (1352–1159 cm): Epiphytic species decline to about 20–50% and number of plankton species increased to 55–65%. Along with the still-prevailing *Lindavia radiosa*, the number of *Pantoseckiella ocellata* and *C. krammerii* increased and new species *Aulacoseira ambigua* appear among the plankton taxa. The number of benthic species also increased slightly, reaching up to 40% in the second half of the zone. Small *Staurosira* and *Pseudostaurosira* species decreased, giving way to benthic *Tabellaria flocculosa*. Some increase in species diversity is noted.

LDAZ-3 (1159–1125 cm): Planktonic species reached up to 65%, although their species composition remained relatively stable, except that species *Aulacoseira ambigua* reduced to a minimum. Epiphytic species are dominated by *Navicula vulpina* and *Gogorevia exilis*. *Tabellaria flocculosa*, *Amphora ovalis* and *Navicula radiosa* prevail among benthic species.

LDAZ-4 (1125–1050 cm): Previously dominant plankton species *Lindavia radiosa* was replaced by *Aulacoseira ambigua* reaching up to 40%. *Staurosira construens* (~45%) and *Navicula vulpina* (~20%) are the most common among epiphypic species. *Navicula radiosa* and *Tabellaria flocculosa* prevail among benthic species.

LDAZ-5 (1050–850 cm): Benthic species reduced from 20 to 5%, and *Tabellaria flocculosa* reduced to minimum; *Geissleria schoenfeldii* and *Navicula radiosa* prevail among them. Planktonic species prevail comprising up to 65% of total sum. *Aulacoseira ambigua* is the dominant species reaching up to 60%, while other plankton species as *Lindavia radiosa*, *Pantoseckiella ocellata* and *Cyclotella cyclopuncta* dissapear in the second part of the zone. Variety of epiphytic species markedly reduced, and *Fragilaria construens* with subspecies prevail reaching up to 60%; *Staurosirella pinnata* comprises up to 10%.

LDAZ-6 (850–750 cm): Plankton and benthic species markedly reduced, and the epiphytic species are the dominant species comprising 80–90% of total sum. *Staurosira construens* ssp. (up to 85%), *Navicula vulpina* (up to 45%), *Staurosira binodis* (up to 20%), *Eunotia arcus* and *Staurosirella pinnata* (up to 5%) are most numerous. *Neidium iridis*, *Stauroneis phoenicenteron*, *Cymbopleura subcuspidata*, *Craticula cuspidata* dominate among benthic species.

### 3.5. Plant Macrofossils Assemblages

**Figure 6:**
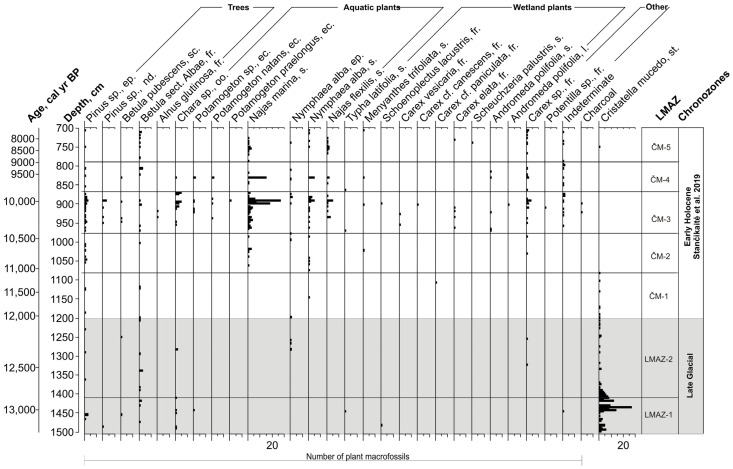
Plant macrofossil diagram of the Čepkeliai section. Zonation above 1200 cm (ČM-1–ČM-5) follows Stančikaitė [34], whereas LMAZ-1–LMAZ-2 represent newly defined local macrofossil assemblage zones. The bar pattern shows the number of recovered plant macrofossils (values indicated on the *X*-axis). Abbreviations: s.—seed, fr.—fruit, ec—endocarp, oo.—oospore, ep.—epidermis, sc.—scale, nd.—needle, l.—leaf, st.—stratoblast.

The plant macrofossil record from the Holocene section (700–1200 cm) has been previously described and illustrated by Stančikaitė [34] and is therefore not discussed further here. This section presents newly analysed plant macrofossil data from the Late Glacial interval (1200–1490 cm). The assemblage includes remains of trees, aquatic plants, wetland plants, and other macroremains. Based on changes in taxonomic composition and abundance, two local macrofossil assemblage zones (LMAZ) were distinguished (Figure 6).

LMAZ-1 (1490–1406 cm): Macrofossil concentrations are low. Aquatic plant remains are represented mainly by *Chara* sp., recorded in most samples. Remains of *Schoenoplectus lacustris* occur sporadically. Macrofossils of *Potamogeton* sp. and *Typha latifolia* appear in the upper part of the zone. Non-plant macroremains are dominated by *Cristatella mucedo* statoblasts, which are abundant in the lower and middle parts of the zone. Tree macrofossils are rare and consist of sporadic occurrences of *Betula* sect. *Albae*, *Betula pubescens* and *Pinus* sp.

LMAZ-2 (1406–1200 cm): Aquatic and wetland plant macrofossils decrease in abundance and taxonomic richness. *Chara* sp. occurs sporadically. *Potamogeton* sp. and *Typha latifolia* are not recorded. *Cristatella mucedo* statoblasts decrease markedly compared to LMAZ-1. Macrofossils of *Nymphaea alba* are recorded in the middle part of the zone. Tree macrofossils remain rare and are represented by isolated occurrences of birch and *pine*.

## 4. Discussion

### 4.1. Late Glacial (13,200–11,650 cal yr BP)

Sediment accumulation at the study site started around 13,200 cal yr BP, which is at the beginning of the Allerød interstadial (GI-1a), dated to 13,200–12,850 cal yr BP. This was one of the most pronounced warming phases of the Late Glacial. Although no independent temperature reconstructions were performed in this study, regional and local proxy-based records suggest that mean July temperatures in Central and Northern Europe generally ranged between 13 and 16 °C [24,55,56,57]. Chironomid-based reconstructions from the Lieporiai site in northern Lithuania indicate that, around 13,300 cal yr BP, July temperatures reached approximately 16 °C [9], whereas lower values (13–14 °C) were reported from Estonia and Latvia [58]. This climatic context provides a valuable framework for interpreting the cladoceran, diatom and macroremain assemblages from the Čepkeliai site, which are highly sensitive to environmental and hydrological changes.

During the initial phase of palaeobasin development, only single diatom frustules were noticed in the sediments. Cladocera assemblages (LCAZ-1) were sparse, dominated by planktonic taxa, primarily *Bosmina longirostris*, with only a few littoral taxa present, indicating limited availability of near-shore habitats. This dominance of planktonic forms suggests relatively deep-water conditions, corroborated by plant macroremains indicating the presence of *Chara* sp. in the basin (LMAZ-1). *Schoenoplectus lacustris* was recorded, indicating the presence of emergent littoral vegetation and relatively stable water conditions [59]. This interpretation of deep-water conditions is further supported by regional data from Lake Varėnis in southeastern Lithuania, where high lake levels and dominance of planktonic diatoms were also recorded around 13,200 cal yr BP [12]. Apparently, during the early stages of its development, the studied palaeobasin was quite deep, with cold and nutrient-poor water. Such conditions were not conducive to the development of cladocerans or diatoms.

A marked shift in environmental conditions occurred around 13,000 cal yr BP (LCAZ-2, LDAZ-1 and the second half of LMAZ-1), as reflected in changes to the composition of all microfossil groups studied. Planktonic cladocera species, including *Bosmina* (*E.*) *longispina*, commonly found in open-water environments [22], disappeared, coinciding with a rise in littoral taxa such as *Eurycercus lamellatus*, *Acroperus harpae*, *Alonopsis elongata*, *Chydorus sphaericus*, and *Pleuroxus trigonellus* (LCAZ-2). This trend indicates a reduction in water depth and a restructuring of available microhabitats. The appearance of *Alonopsis elongata*, typically associated with cold waters [60,61], may suggest continued influence of cooler conditions. Simultaneously, the occurrence of *Daphnia* sp. (especially the *Daphnia pulex* group) and *Bosmina* (*E.*) *longispina*, taxa often found in deep, oligotrophic northern or alpine lakes [62,63,64], points to persistent low water temperatures and still relatively well-developed pelagic conditions during this transitional period.

Water level fluctuations are also reflected in the composition of diatoms, with the number of planktonic species ranging from 2 to 30% (LDAZ-1). However, the predominance of benthic diatom species indicates a relatively low water level. The optimal water transparency (SD) tolerated by the most abundant benthic species such as *Pseudostaurosira brevistriata*, *Staurosira construens* and *Staurosirella pinnata* ranged from 3.6 to 1.3 m [33]. Regional data from Varėnis also indicate a gradual water level decline between 13,200 and 12,600 cal yr BP, inferred from a slight increase in epiphytic diatoms. This pattern mirrors the transition observed at the Čepkeliai site from planktonic dominance to greater littoral diversity, suggesting that lake shallowing was part of a broader regional trend [12]. Dominant *Staurosira construens* spp. have been referred to as “pioneers: as they are often the first colonisers of recently deglaciated sites and are not demanding in terms of environmental conditions [65,66,67]. The dominant epiphytic species *Pseudostaurosira brevistriata* is considered an indicator of oligotrophic conditions; however, it can also occur in slightly more nutrient-rich environments than those typically found in alpine lakes [33,68,69,70]. Also, it is often abundant in shallow, meso-eutrophic waters [71]. Taken together, Fragilariaceae species indicate an oligotrophic, oxygen-rich and alkaline lacustrine environment [48,72]. Despite the increase in littoral species, the overall concentration of Cladocera remained low. This pattern likely reflects early colonisation stages, with underdeveloped macrophyte cover, limited primary productivity, and cool water temperatures constraining community development and biomass accumulation. Notably, an increase in *Pleuroxus trigonellus* and the appearance of *Pleuroxus uncinatus* stenothermal taxa preferring warm, nutrient-rich waters [73,74] suggests a subtle warming trend and enhanced nutrient availability. These changes may be attributed to increasing runoff or early shoreline vegetation expansion, facilitating the development of more productive littoral environments. The synchronous rise in *Cristatella mucedo*, a bryozoan species indicative of shallow, moderately warm waters with elevated nutrient levels [75,76], supports this interpretation.

Macrofossil data confirm this ecological shift, indicating the spread of *Potamogeton* sp. and *Typha latifolia* within the basin (LMAZ-1). The occurrence and increasing abundance of *Cristatella mucedo* imply water depths around 2 m, temperatures not lower than 11–16 °C, reduced wave activity, moderate to high calcium and magnesium concentrations and slightly acidic pH, conditions conducive to bryozoan colonisation [75,76]. While aquatic vegetation remained relatively sparse, the co-occurrence of bryozoans and littoral cladocerans suggests the emergence of submerged macrophytes, providing suitable substrates and promoting habitat complexity. The appearance of *Typha latifolia*, a species typically associated with shallower water and warmer climates, further supports the interpretation of progressive warming and lake shallowing during this interval. *Typha latifolia* thrives where mean July temperatures range between 13 and 16 °C and is indicative of low water levels [77,78].

In summary, the initial phase of palaeobasin development (Allerød) at the study site was marked by a transition from deep, cold-water conditions toward a shallower, warmer, and more ecologically diverse littoral system. The palaeobasin was apparently oligotrophic–mesotrophic, which is confirmed by the relatively high content of terrigenous matter (~60%) (Figure 2). This transformation was driven by gradual climatic warming and accompanied by increasing aquatic productivity and macrophyte colonisation, as reflected in microfaunal, diatom, and plant macroremain data.

Further noticeable changes in the palaeoenvironmental development occurred around 12,850 cal yr BP. Cladocera abundance was low (end of the LCAZ-2), suggesting that conditions were not favourable for their development. The basin was shallow and dominated by a limited number of planktonic taxa, such as *Bosmina* (*E.*) *longispina* and *Bosmina* (*E.*) *coregoni*, with *Daphnia pulex* notably absent. Cold-tolerant taxa, including *Acroperus harpae*, *Eurycercus lamellatus*, *Alona nana*, *Sida crystallina*, and *Chydorus sphaericus*, predominated. These species are typically associated with low temperatures, harsh abiotic regimes, and nutrient-poor environments. Cold environmental conditions are proven by the presence of a relatively rare taxon—*Alonopsis elongata*—characteristic of northern Europe. Similar summer temperatures (~11–11.5 °C) have been reconstructed at contemporaneous sites including Nakri (Estonia), Kurjanovas (Latvia), Kamyshovoye (southern Baltic), and Petrašiūnai (Lithuania) [5]. An increase in *Graptoleberis testudinaria* may indicate the expansion of shoreline macrophytes [16], although this is not corroborated by macrofossil data, possibly due to poor preservation under unfavourable taphonomic conditions.

These palaeoenvironmental changes appear to be linked to the onset of the Younger Dryas (12,850–11,650 cal yr BP), a major cold reversal marking the final stage of the Late Glacial. Existing regional data clearly reflect a significant cooling trend. In northeastern Lithuania, pollen-based reconstructions from the Petrašiūnai site suggest mean July temperatures between 11.1 and 11.4 °C [5], while chironomid-inferred values from the Lieporiai site reached around 14 °C [9]. Similar or lower values were recorded across central and northern Europe including 12 °C at the Żabieniec site [57] and 9–10 °C in Norway and Switzerland [79].

Around 12,600 cal yr BP (LCAZ-3), a rise in lake level is inferred from the increasing abundance of planktonic cladocera taxa, likely reflecting reduced evapotranspiration associated with colder climatic conditions [80]. The higher water level is proven by a marked increase in planktonic diatom species (LDAZ-2), dominated by *Lindavia radiosa*, *Pantocsekiella ocellata* and *Cyclotella krammeri*. These are cosmopolitan species that are often found in oligotrophic and relatively deep high-transparency lakes, particularly those of sandy and gravel lake beds [81,82], and this is confirmed by the significant amount of terrigenous material (~40%) (Figure 2). The species *Tabellaria flocculosa*, which is prevalent in benthos, points to low-nutrient soft water [83]. A significant number of benthic taxa, accounting for up to 30%, indicate clear water conditions. Climate cooling led to the emergence of species *Navicula vulpina* which appears to have a circumboreal distribution as suggested by Antoniades [84]. At the end of this cooling, about 11,500 cal yr BP, the species *Gogorevia exilis*, which is prevalent in cool, alkaline waters, peaked and then disappeared.

This hydrological trend is consistent with a well-documented regional pattern observed across Central and Eastern Europe during the Younger Dryas. In Lake Varėnis (southeastern Lithuania), a decline in epiphytic diatoms and the dominance of planktonic forms between 12,600 and 11,200 cal yr BP indicate a sustained increase in water depth despite the lack of distinct lithological changes [12]. Similar lake-level rises attributed to climate-driven humidity increase and reduced evaporation were reported from Lake Linówek in Poland [85], Lake Gościąż [86], and two lakes in southeastern Lithuania [87]. In Belarus, elevated lake levels were noted in the first half of the Younger Dryas (12,900–12,000 cal yr BP) in multiple basins, including Lozoviki, Mezhuzhol, Sudoble, and Oltushskoe, reflecting a regional increase in atmospheric moisture [88].

In the studied basin, these hydrological changes were associated with the development of oligo-mesotrophic conditions, an expansion of open-water zones, and the increasing dominance of *Bosmina* (*E.*) *coregoni* and *Bosmina* (*E.*) *longispina* (LCAZ-3). The prevalence of *Eubosmina* and the continued presence of *Alonopsis elongata* point to cool waters and greater basin depth [61]. A concurrent decline in *Cristatella mucedo* likely reflects the submergence of shallow littoral habitats caused by lake-level rise. The dominance of *Bosmina* (*E.*) *longispina*, a species typical of cold, low-carbonate waters [23], further supports the interpretation of oligotrophic, deep-water conditions in the basin [89]. A marked decrease in *Alona rectangula*, a species preferring nutrient-rich waters, coincides with increased frequencies of *Bosmina* (*E.*) *longispina* and *Bosmina* (*E.*) *coregoni*, species associated with waters of a lower trophic level [90]. Reductions in *Eurycercus lamellatus*, *Acroperus harpae*, *Alonella nana*, *Chydorus sphaericus*, and *Graptoleberis testudinaria* further support this interpretation. Conversely, an increase in *Chydorus pigra* and *Alonopsis elongata*, both considered indicators of clear-water, low-nutrient environments [17,73], suggests high water transparency. The presence of *Chydorus pigra* may also reflect slightly acidic water, a condition supported by the appearance of *Alona excisa*. The oligotrophic–mesotrophic water conditions are also indicated by the relatively high content of terrigenous material, fluctuating between 40 and 60% with an overall increase upwards.

In summary, during the Younger Dryas period, the water level in the palaeobasin rose. This interpretation is supported by the general correspondence between the cladocera (LCAZ) and diatom zones (LDAZ) during this interval. The water remained cold and fairly clear but still lacked sufficient nutrients. Although cold-adapted taxa dominated the assemblage, some thermophilous forms, such as representatives of the cladocera genus *Pleuroxus*, were also recorded. This suggests the presence of locally favourable summer conditions and shallow littoral habitats during the Younger Dryas. Similar patterns have been noted in Polish lakes [91,92] and other parts of Europe [93,94]. The continued presence of *Cristatella* statoblasts suggests that summer temperatures likely did not fall below 10 °C [75].

Around 12,000 cal yr BP (LCAZ-4), a decline in lake level occurred, accompanied by an increase in littoral taxa of Cladocera. Meanwhile, the diatom flora showed no signs of a drop in water level, though there may be a slight decrease in the abundance of epiphytic species (LDAZ-3). The cold-adapted *Alonopsis elongata* disappeared, while *Camptocercus rectirostris* and *Alonella excisa* became more abundant. *Camptocercus rectirostris* is typically associated with improved climatic conditions and warmer waters [95]. These changes are supported by the macrofossil record (the end of the LMAZ-2), which documents the disappearance of *Chara* and the appearance of *Nymphaea alba*, a species indicative of mean July temperatures above 12 °C [96]. Evidence for a temperature increase near the end of the Younger Dryas has also been reported from other European lakes [93,97,98,99]. The warming, which began approximately 100–150 years before the onset of the Holocene, appears to have been more pronounced in the East European Plain than in Scandinavia or alpine regions [100].

### 4.2. Early Holocene (11,650–8200 cal yr BP)

The Early Holocene (11,650–8200 cal yr BP) in northeastern Europe was marked by a gradual climatic warming following the Younger Dryas. While global temperature rise began shortly after 11,500 cal yr BP [101], the onset of warming was slightly delayed in continental regions compared to the North Atlantic coast [102,103]. In the Baltic region, including Lithuania, warming trends are evident in sediment sequences from around 11,100 cal yr BP [6,13]. Chironomid-based reconstructions indicate that mean summer temperatures during this period reached approximately 13.3 °C [5]. These conditions likely shaped the ecological succession observed in the Čepkeliai basin, particularly in the composition and abundance of Cladocera, plant macrofossils, and diatoms.

At the onset of the Early Holocene, around 11,650 cal yr BP, Cladocera species’ increased richness and abundance suggest warm and relatively humid climatic conditions in the basin (end of LCAZ-4; LCAZ-5). The prevailing environmental settings were favourable for Cladocera development. The assemblage included both cold-adapted taxa (e.g., *Chydorus sphaericus*, *Acroperus harpae*, *Eurycercus lamellatus*, *Alonella nana*, *Alona affinis*) and thermophilous forms (e.g., *Pleuroxus trigonellus*, *Camptocercus rectirostris*, *Sida crystallina*), reflecting a moderately warm climatic regime during this interval [104]. The high abundance of *Camptocercus rectirostris*, a species that prefers warmer waters, alongside the presence of *Sida crystallina* and *Pleuroxus trigonellus*, confirms improving thermal conditions. The dominance of littoral taxa supports the interpretation of a shallow basin, consistent with previous studies [34].

Numerous palaeoenvironmental studies have documented a lowering of lake level during the Early Holocene in various regions including Poland [85,105], Belarus [88], Latvia [106], Lithuania [13,107], and Scandinavia [108]. The presence of macrophyte-associated species such as *Acroperus harpae*, *Alona affinis*, *Alonella nana*, and *Eurycercus lamellatus* indicates a well-developed littoral vegetation. The co-occurrence of taxa typical of both oligo-mesotrophic and eutrophic environments suggests mesotrophic to eutrophic conditions in the lake. The occurrence of *Chydorus pigra* may be linked to low pH conditions, as this species prefers slightly acidic, oligotrophic or dystrophic waters, including shallow *Sphagnum*-dominated pools in peatlands [109,110]. Its expansion may signal the initial stages of peat formation near the basin.

This transitional period is characterised by significant changes in diatom composition. The previously dominant planktonic species, the oxygen-demanding *Lindavia radiosa*, was replaced by *Aulacoseira ambigua*. This species is prevalent in mesotrophic-eutrophic waters and favours turbid conditions. Lower water transparency is also indicated by the reduced number of benthic species and the increased abundance of epiphytic species. Although the number of epiphytic species remains similar, their diversity is significantly reduced, which may also be due to increased water-mixing processes. The epiphytic *Staurosira construens* dominates again. It is a widely distributed diatom species found in various freshwater habitats. This variability highlights the species’ adaptability to different trophic states. In the study of the Central European Diatom training set, this species was predominantly found in lakes with low transparency and elevated nutrient concentrations [33]. All of these changes indicate an intensification of the eutrophication process. This is also evidenced by the increased organic matter content of 70–80% (Figure 2).

Around 10,645 cal yr BP (LCAZ-6), an increase in *Chydorus sphaericus* abundance proves a rise in the trophic level, indicated by diatoms. Many authors associate the proliferation of this taxon with eutrophication and a shift toward higher productivity [111,112]. Simultaneously, a decline in *Camptocercus rectirostris* and an increase in *Alona rectangula*, *Leydigia acanthocercoides*, and *Pleuroxus uncinatus* further reflect increasing trophic status [113]. The reduction in *Bosmina* (*E.*) *longispina* abundance also supports a eutrophication trend [114]. An increased presence of *Najas marina* during this period (ČM-2, ČM-3), a species favouring eutrophic waters, provides additional evidence of rising trophic levels. Around 10,200 cal yr BP (end of LCAZ-6), an increase in planktonic taxa indicates a water level rise likely associated with a wetter climate [34]. By approximately 10,000 cal yr BP, *Chydorus sphaericus* declined sharply, likely reflecting changing environmental conditions. This is also reflected by the sharp decline in the diversity of diatom species around 9700 cal yr BP (LDAZ-6), when planktonic species almost became extinct. The sediments are dominated by small epiphytic representatives of the *Staurosira* genus. The small size of *Staurosira* (*Fragilaria*) communities and their high reproductive rate as well as large ecological amplitude characterise them as species that can adapt to the changing environmental conditions and can be a good indicator of high environmental stress, physical disturbance, and unstable transient conditions [115]. Approximately 90% of the diatom flora consists of epiphytic species. The epiphytic diatom *Eunotia paludosa* is an indicator of dystrophic and low pH environments [116]. The organic matter content varies between 80 and 90%. These findings suggest the onset of palaeobasin swamping processes.

Changes in the composition of cladocera also indicate the same palaeoenvironmental conditions. Between 9200 and 9000 cal yr BP (beginning of LCAZ-8), the abundance of thermophilous taxa such as *Bosmina longirostris*, *Pleuroxus trigonellus*, and *Alona guttata* decreased, possibly reflecting a short-lived climatic cooling event. This assumption is confirmed by a short-term peak in planktonic diatom species (up to 30%), which indicates a rise in water level, and the appearance of the boreal species *Navicula vulpina* (LDAZ-6). After 9000 cal yr BP, *Graptoleberis testudinaria* appeared and *Camptocercus rectirostris* increased, changes that may be associated with temperature rise, as these species are typically found in warmer waters [104]. During this period, water temperatures were evidently high enough to support the development of both *Pleuroxus trigonellus* and *Graptoleberis testudinaria* [117]. The latter species also indicates an increased content of insoluble organic matter and is often found in peatland environments [109]. The appearance of *Alonella excisa* and *Alona exigua* may be associated with decreased productivity, dense macrophyte growth, and low pH conditions [112]. These species typically occur in acidic waters dominated by *Sphagnum* vegetation [118]. Such water conditions are also evidenced by the increased abundance of *Alonella excisa*, a species typical of humic waters [119]. During this period, a decline in taxa associated with fertile waters—such as *Bosmina longirostris* and *Leydigia acanthocercoides*—was recorded, indicating a decrease in trophic status. This process is supported by the reduction in *Alona guttata*, considered a reliable eutrophication indicator [104]. The decrease in planktonic species indicates a drop in water level. The abundant presence of *Acroperus harpae* and *Alona affinis* points to dense aquatic vegetation in the littoral zone [120]. *Chydorus pigra* was also recorded at this in time, a species associated with slightly acidic, oligotrophic, or dystrophic waters, particularly in *Sphagnum*-dominated bog pools [109,110]. An increase in *Camptocercus rectirostris*, *Alona affinis*, and *Alonella excisa* abundance may reflect further expansion of the macrophyte zone. The dominance of Cladocera taxa preferring vegetated habitats (e.g., *Alona affinis*, *Camptocercus rectirostris*) suggests a reduction in overall basin size. The presence of *Graptoleberis testudinaria* may be associated with an increased input of dissolved organic matter, particularly from plant detritus [109].

At approximately 8139 cal yr BP, an increase in nutrient availability is inferred from the elevated abundance of *Alona rectangula* and *Chydorus sphaericus* in the sediment record. The concurrent rise in thermophilous taxa such as *Pleuroxus trigonellus* and *Sida crystallina* likely reflects climatic warming. Despite these changes, the water level remained relatively low.

## 5. Conclusions

Recent Cladocera, diatom and plant macrofossil studies at Čepkeliai peatbog provided the opportunity to reconstruct the Late Glacial and Early Holocene environmental history of the site. The initial phase of the palaeobasin development (13,200–13,000 cal yr BP) was marked by deep, oligotrophic and cold-water conditions and can be correlated with the GI-1b event (Gertsenzee oscillation). A significant ecological shift towards a shallower, warmer and more ecologically diverse littoral system was observed at around 13,000 cal yr BP. This transformation was driven by gradual climatic warming and accompanied by increasing aquatic productivity and macrophyte colonisation and is consistent with the GI-1a (Allerod) warming period. The beginning of the 12,850–11,650 cal yr BP time period (GS-1; Younger Dryas) is characterised by a rise in lake level and development of oligo-mesotrophic high water-transparency conditions. The existence of thermophilic species alongside cold-adapted taxa indicates that environmental conditions were not stable throughout the entire period. At the end of the Younger Dryas, around 12,000 cal BP, an improvement in climatic conditions and a drop in water levels were recorded in changes in the composition of Cladocera. Meanwhile, significant changes in diatom flora are observed around 11,500 cal BP, at the beginning of Preboreal. This transitional period is characterised by improved climatic conditions that foster a shallow environment with low water transparency and a tendency towards eutrophication. Intensive palaeobasin swamping processes began around 9700 years BP (Boreal), as evidenced by changes in the fauna and flora studied and the increasing amount of organic matter. Changes in the composition of Cladocera and diatom taxa allowed us to record a short-lived “9.2” cooling event at about 9200–9000 cal yr BP. The data obtained show that the use of several biological proxies for palaeoenvironmental reconstruction is highly complementary and significantly enhances the information provided by each proxy, resulting in more reliable interpretations. The results of the environment reconstruction correlate perfectly with previous studies conducted in Lithuania and neighbouring areas, adding to our knowledge of postglacial palaeoenvironmental dynamics in the southeastern Baltic region.

## Figures and Tables

**Table 1:** Lithological makeup of the Čepkeliai core.

Depth, cm	Lithology
700–800	Gyttja, greenish brown
800–1034	Gyttja, greenish, dark brown
1034–1050	Gyttja, greenish, dark brown, with white layers
1050–1100	Gyttja, light greenish
1100–1200	Gyttja, calcareous, light greenish, silty and darker at the bottom
1200–1300	Dark greenish grey silty gyttja
1300–1370	Dark greenish-grey silty gyttja with black layers
1370–14,100	Black gyttja
14,100–15,000	Sandy black gyttja

**Table 2:** Results of radiocarbon (^14^C) dating. Radiocarbon dates from 700 to 1200 cm correspond to the Holocene section and were previously published by Stančikaitė [34].

No.	Depth, cm	Dated Material	Lab. Index.	^14^C Age	Cal Age (BP)	
					1 σ (68.2%)	2 σ (95.4%)
1	790–794	TOC	Vs-2642	8065 ± 140	9140–8700 (67.3%) 8670–8660 (0.9%)	9400–9350 (2.2%) 9325–8590 (93.2%)
2	832–836	TOC	Vs-2643	8850 ± 215	10,195–9665 (68.2%)	10,495–10,450 (1.0%) 10,440–9480 (94.4%)
3	1034–1038	TOC	Vs-2646	9355 ± 205	11,065–11,030 (2.8%) 10,995–10,970 (2.0%) 10,790–10,255 (63.5%)	11,205–10,175 (95.4%)
4	1194–1198	TOC	Vs-2647	10,205 ± 240	12,380–12,325 (3.7%) 12,310–12,270 (2.3%) 12,240–11,595 (54.2%) 11,555–11,470 (5.8%) 11,445–11,405 (2.3%)	12,595–11,225 (95.4%)
5	1248–1252	TOC	Vs-2893	10,510 ± 195	12,660–12,140 BP	12,765–11,745 BP
6	1364–1366	TOC	Vs-2652	10,720 ± 280	12,980–12,370 BP	13,190–11,760 BP
7	1402–1408	TOC	Vs-2894	10,960 ± 195	13,030–12,710 BP	13,250–12,550 BP
8	1444–1446	TOC	Vs-2653	11,015 ± 355	13,290–12,590 BP	13,630–12,030 BP

## Data Availability

The data are stored at the State scientific Research Institute Nature Research Centre and are available on request.
